# Surgical Cases

**Published:** 1850-07

**Authors:** Charles Sutherland

**Affiliations:** Late Assistant to the Clinic of Jefferson Medical College


*Surgical Cases.* Reported by CHARLES SUTHERLAND, M. D., late Assistant to the Clinic of Jefferson Medical College.

The following have been selected from a number of cases which presented themselves at the Clinic of Jefferson Medical College.

A man, aged 45 years, presented himself last fall with a large tumor upon his right side. It had been growing for eight years past, and it now gave rise to a great deal of pain and inconvenience. It was perceptibly enlarging, and he resolved to have it removed. It extended from within a few inches of the inferior angle of the scapula to the floating ribs, and from within an inch of the vertebræ up to and under the axilla. The tumor was evidently under the latissimus dorsi muscle, and it felt hard, with soft spots here and there throughout its whole extent. Dr. Mütter was of the opinion that it was of the sero-cystic variety. On January the 16th he was brought before the class, and the operation performed by Dr. Mütter, assisted by Dr. Pancoast.

The first incision extended from the upper to the lower part of the tumor, in the median line, about eight inches in length, and then from the middle of this incision back towards the spine, being about four inches in length. A T-shaped wound was thus made, the knife in this stage of the operation passing through skin and fascia. The operator then proceeded to dissect off the flaps, which was soon accomplished. One or two arteries were tied. The fibres of the muscle which covered the tumor were next cut through, the incision in this case being similar to the one first made. Several arteries were ligated. The tumor was torn out of its place in a gradual manner, occasionally using the knife where it was required to divide some adhesive bands. The object in thus removing it was to effect the laceration of the capillary vessels, and thus prevent secondary hemorrhage. After working above and below in the manner described, the pedicle of the tumor was reached. Near to its base, which extended a considerable distance up into the axilla, coursed the axillary artery, which could be seen distinctly pulsating. The subscapular artery which was enormously dilated, ran through the pedicle into the tumor, and most of the arteries which were tied came off from this large branch of the axillary. Around the pedicle was passed a strong double ligature, which being firmly tied, arrested all vascular communication. After this was accomplished the attachment was severed and the tumor removed. It weighed after its extirpation $10\frac{1}{2}$ pounds, and proved upon examination to be a most perfect specimen of the sero-cystic variety. There was some little oozing, but this ceased after tying several small arteries, and when the coating of plasma had fully appeared. Soon after, the flaps were brought together. It might be supposed that they would more than cover the wound, but this was not the case; they had sufficiently retracted to unite evenly when brought into apposition with each other. They were maintained in their proper position by sutures, adhesive straps, and a firm bandage, which was applied around the chest. The patient was immediately put to bed. A few hours subsequently some oozing came on, consequent upon reaction; towards night it was completely arrested. A teaspoonful of Liq. Morph. Sulphat. was given to him every hour or so. His sleep the first night was restless and disturbed: he complained of pain in his right shoulder, which also extended down his arm. The day after he was more comfortable; pain not so severe. A light diet was ordered. Remained in this condition for several days. On the fifth day after the operation the dressings were removed. The transverse cut, together with the upper and lower part of the long incision, had all united, save where they met in the centre. Here suppuration was going on to a slight extent, partly owing to the ligatures which came out at this place. The part was again dressed, and a flaxseed poultice applied. It was afterwards found necessary to dress the wound twice a day. Under this local treatment, combined with a tonic regimen, the man daily improved, and was able a week after the operation to go about his room, and a week from that period he walked into the college and exhibited himself to the class. Soon after all the ligatures came away, the suppurating portion healed by granulations from the bottom, hastened by a stimulating ointment; the patient's health greatly improved and finally during the early part of March he was discharged cured.

The following is a brief account of a case of mortification attributable to severe and continued cold. The sufferer was a German lad, aged about twelve years, who worked upon a farm in the lower part of the State of New Jersey. One day last winter, for some slight dereliction of duty, he was severely chastised by his master, and on the following was confined in a barn upon the place, where he was forced to remain all night, which was one of the coldest of the season. His clothing was scanty, and, as might be supposed, he suffered extremely from the effects of the cold. On the following morning he was found unable to stand erect, there being total loss of sensation and motion in his feet. This was followed by an active throbbing pain, redness and swelling. Effusion occurred to a great extent in the cellular tissue. After this followed mortification. Both feet appeared to be dead, the right foot seemed the worst of the two. During this time he was judiciously treated by a physician residing in the neighborhood, but all his efforts proved unavailing, and the lad was sent to this city for the purpose of having his feet amputated. When he arrived at the hospital of the Jefferson Clinic, (on the 11th Feb.) he had upon him only a shirt, a thin pair of pants, and a vest, and was covered by a small blanket. The patient was depressed in spirits, complained of pain in several parts of his body, and also of cold.

On examining the feet, the entire sole and dorsum of one (the right foot) was found to be completely mortified; the sole of the other was sphacelated nearly throughout the extent of its surface, and on its front as far as the tarso-metatarsal joint. A line of healthy granulations divided the living from the dead parts in both feet, better marked however in the left one. The sloughs were of a dark ash color and intolerably offensive. The boy was put to bed, warm clothing placed on and around him, he partook of some broth which revived him somewhat, at night a purgative was administered, and the feet enveloped in large poultices composed of carrots and powdered charcoal.

On the 12th, Did not sleep much during the night, bowels opened, feet again dressed with same applications. Dr. Pancoast now saw the case for the first time.

13th. After consultation it was resolved to amputate the right foot, and leave the left one, in which the dead mass showed a disposition to fall off, in which case, if it did, the tarsal bones would remain un-involved. Accordingly on the same day the right foot was removed by amputating above the ankle in the lower third of the leg. Dr. Pancoast performed the flap operation and obtained a capital stump.

14th. Patient very comfortable; recovered from effect of the operation admirably; the slough of the remaining foot ready to fall off; seems to adhere by means of two metatarsal bones.

17th. The dead mass came away with the dressing; two metatarsal bones of second and third toes alone remained. Dressing changed, flaxseed now used.

18th. Exhibited to the class, stump dressed first time before them. It had united by the first intention. Dr. Pancoast intended to remove the projecting bones of the left foot, but the boy, from some reason or other, just before he was brought to the college, wrenched the bones out their places. Some diseased portions of bone were removed by the bone forceps. From that time forward, cicatrization proceeded rapidly in this foot. The ligatures came away soon after.

22d. Able to walk about the room by means of a crutch.

March 1. Both stumps entirely healed. By means of an artificial foot and ankle, and the support that he will obtain from the tarsal bones, the boy will be enabled to walk tolerably well. In a few days subsequently he was discharged cured. He is now learning the trade of a tailor.

A colored lad, æt. 15 years, had been laboring for some time under white swelling of the knee-joint as a consequence of improper treatment. Great contraction of the tendons of the hamstring muscles ensued. The limb was flexed at nearly a right angle, and while in this position permanent anchylosis of the joint was the result. The limb consequently was rendered useless, and after consultation it was determined to remove it by amputating through the thigh. On the afternoon previous to the day fixed upon for the operation, the patient incautiously stepping from his bed, fractured the shaft of the femur of the diseased side. The effect of this was to prostrate him severely. He was brought to the college, and primary amputation would have been performed, had sufficient reaction occurred. On the subsequent day he had reacted so slightly as not to warrant a removal of the limb. Diffusive stimuli were freely used, and it was not until after persisting in their use for several days, that the patient's system was brought up to the point desired. The fracture, however, was set—four short splints applied around the thigh and the limb rested upon an inclined plane of pillows. The boy was brought into the college upon the next clinic day. Every thing was favorable, and the limb was removed by Dr. Mütter. A question arose at the time, as to the propriety of amputating through the seat of fracture or above it. The first plan was preferred; the callus being soft and yielding, there would be no difficulty in passing through it, and would save the necessity of sawing the bone. If any spicula of bone existed, they would be removed by means of the bone nippers. The flap operation was performed. The ends of the bone were found surrounded by a soft provisional callus; a fine stump was obtained after ligating the blood vessels, and waiting for the formation of the coat of the plasma. The flaps were brought together, united according to the usual mode, and the cold water dressing applied. The patient was put to bed, and no secondary hæmorrhage followed. Suffice it to say, the subsequent treatment was upon general principles, the flaps united by the first intention, and in less than three weeks the boy was discharged cured.

A large fatty tumor was removed, last February, from the thigh of a patient, by Dr. Pancoast. It had been slow, but progressive in its growth, and was situated on the inside of the left thigh, about five or six inches from the pubis. It was a lobulated, softish, and painless tumor, feeling like a mass of fat. It was in length some seven or eight inches; and in width five inches, save at its pedicle, where, it was not more than two inches. The patient, a man, was placed under the influence of ether. A straight incision was made, extending from the upper to the lower part of the tumour; passing through the skin, and the cyst of cellular tissue, which surrounded it. After which it was rapidly removed; principally, by tearing its cellular connections. The tumor consisted of lobulated masses of fat, and possessed little vascularity; no ligature was required. The fatty mass was removed completely; only a solitary lobule was broken, which was taken away entirely. The tumor, if it had been allowed to remain, for a longer time than it did, might have taken upon itself a malignant character. This was partly shown, by examining one of its smallest lobules, which was already undergoing an unfavorable change in its condition. The part was treated as an incised wound, adhesive plasters maintained the parts in situ, and a spica bandage, kept all snug. In less than two weeks from that time the patient was well.

